# Effect of heat stress on blood biochemistry and energy metabolite of the Dazu black goats

**DOI:** 10.3389/fvets.2024.1338643

**Published:** 2024-05-27

**Authors:** Le Wang, Pengjun Zhang, Yuxuan Du, Changtong Wang, Li Zhang, Li Yin, Fuyuan Zuo, Wenming Huang

**Affiliations:** ^1^College of Animal Science and Technology, Chongqing Beef Cattle Engineering Technology Research Center, Southwest University, Chongqing, China; ^2^Chongqing Academy of Animal Sciences, Chongqing, China; ^3^Chongqing Animal Husbandry Technology Extension Station, Chongqing, China

**Keywords:** heat stress, Dazu black goat, physiological indicators, hormonal, amino acid metabolism

## Abstract

The objective of this study was to determine the effects of heat stress (HS) on physiological, blood biochemical, and energy metabolism in Dazu black goats. Six wether adult Dazu black goats were subjected to 3 experimental periods: high HS (group H, temperature–humidity index [THI] > 88) for 15 d, moderate HS (group M, THI was 79–88) for 15 d, and no HS (group L, THI < 72) for 15 d. Rectal temperature (RT) and respiratory rate (RR) were determined on d 7 and 15 of each period, and blood samples were collected on d 15 of each period. All goats received glucose (GLU) tolerance test (GTT) and insulin (INS) tolerance test on d 7 and d 10 of each period. The results showed that HS decreased dry matter intake (DMI) and INS concentration (*p* < 0.05), and increased RT, RR, non-esterified fatty acid (NEFA), cortisol (COR), and total protein (TP) concentrations (*p* < 0.05). Compared to group L, the urea nitrogen (BUN) concentration increased and GLU concentration decreased in group H (*p* < 0.05). During the GTT, the area under the curve (AUC) of GLU concentrations increased by 12.26% (*p* > 0.05) and 40.78% (*p* < 0.05), and AUC of INS concentrations decreased by 26.04 and 14.41% (*p* < 0.05) in groups H and M compared to group L, respectively. The INS concentrations were not significant among the three groups (*p* > 0.05) during the ITT. A total of 60 differentially expressed metabolites were identified in response to groups H and M. In HS, changes in metabolites related to carbohydrate metabolism and glycolysis were identified (*p* < 0.05). The metabolites related to fatty acid β-oxidation accumulated, glycogenic and ketogenic amino acids were significantly increased, while glycerophospholipid metabolites were decreased in HS (*p* < 0.05). HS significantly increased 1-methylhistidine, creatinine, betaine, taurine, taurolithocholic acid, inosine, and hypoxanthine, while decreasing vitamin E in blood metabolites (*p* < 0.05). In summary, HS changed the metabolism of fat, protein, and energy, impaired GLU tolerance, and mainly increased amino acid metabolism to provide energy in Dazu black goats.

## 1 Introduction

Heat stress (HS) is the body’s nonspecific response under high temperature environment to produce a reaction combined. Animals with HS may experience a range of physiological and behavioral abnormalities that impair their ability to reproduce, grow, and produce (1, 2). According to a study by Hashem et al. (3), HS reduced the pH, cooking loss, water holding capacity, and shear force of Black Bengal goats’ meat. HS has been shown to decrease growth performance and milk production of dairy goats by 12 and 3–10%, respectively (4, 5). HS can result in total animal losses averaging $2.4 billion annually (6). In addition, HS may cause damage to animal proteins, fats, and carbohydrates metabolism (7). It was found that HS caused a significant decrease in the abundance of several polar lipids such as phosphatidylcholine, phosphatidylserine, lysophosphatidylcholine, and glucosylceramide, while significantly increased the activities of glycogen phosphorylase and pyruvate dehydrogenase in muscle, as well as increased protein degradation (8, 9). The temperature–humidity index (THI) is a common bioclimatic indicator to assess HS. Goats critical temperature of the HS in 35–40°C (5), and goats can occur when the THI exceeds 80 (10). Goats have a wide isothermal zone, are highly resistant to heat (11), and recover from HS via physiological, biochemical, and metabolic changes (12–14). However, HS and even death of goats may occur at high environmental temperature (ET) and relative humidity (RH), especially for goats with production needs. ET of 34–36°C are reported to reduce the conception rate of female goats (15) and HS was found to decrease the expression of genes related to reproductive efficiency in Malabari goats (16). The Dazu black goats are native to Chongqing, China, and are characterized by a short black coat. They have the characteristics of cold and drought tolerance, strong stress resistance, efficient disease resistance, and roughage resistance, which are of great significance to the development of livestock farming. Notably, black coats absorb more solar radiation, and short-haired goats tolerate radiant heat less than hairy goats (5). In Chongqing, the ET exceeds 35°C for an average of 40.77 d during the summer months with an average annual RH of 80%, which belongs to the high humidity area. Moreover, the housing environments of goats can be even more humid, which is more likely to cause HS for Dazu black goats. To sum up, no HS occurs when THI is less than 77.33 (17), was alert and in danger between 80 and 90, with extreme danger beyond 90 for goats (10), which influenced the physiology, blood biochemical indices, metabolism, and even cause the death of goats. The performance of the Dazu black goats were greatly affected in the summer. Therefore, it is of great significance to study the changes in physiological indexes, blood biochemical indexes, and blood metabolites of Dazu black goats to improve the metabolic regulation, feed digestibility, and growth performance of goats during HS in order to prevent and control HS.

To date, relatively few studies have investigated the physiological and metabolic changes of Dazu black goats in response to high ET and RH. Therefore, the present study aimed to assess the effects of HS on the physiological and blood biochemical indices as well as endogenous metabolites of Dazu black goats. The results of this study will help to clarify the response mechanisms of goats to HS and provide basic parameters for efficient and healthy breeding under hot and humid conditions.

## 2 Materials and methods

### 2.1 Animals, diets, and experimental design

Six wether adult Dazu black goats (28.4 ± 3.2 kg of body weight) were subjected to 3 experimental periods with a single-factor self-controlled trial. The total length of the trial was 52 d, with the pre-feeding period was 7 d and the 3 experimental periods consisting of (1) 15 d of high HS (group H, THI > 88); (2) 15 d of moderate HS (group M, THI was 79–88); and (3) 15 d of no HS (group L, THI < 72). ET and RH were manually controlled for temperature and humidity with the use of 4 heaters, 2 humidifiers, and 2 air conditioners. Three temperature and humidity data logger devices (Testo, Inc., Sparta Township, NJ, USA), which located about 1.6 meters above the ground, were used to record the ET and RH every 30 min. The ET and RH were under control from 8:00 to 18:00 h and 18:01 to 7:59 h the next day during the three treatment phases. THI was calculated from ET and RH values which were recorded. Table 1 displays the ET, RH, and THI during the duration of the experiment. The THI was calculated according to the formula described in Hamzaoui et al. (18):


$$\text{THI}=\left(1.8\times T_{\text{db}}+32\right)–\left(0.55–0.0055\times \text{RH}\right)\;\left(1.8\times T_{\text{db}}–26.8\right)$$

where T_db_ is the dry bulb temperature (°C) and RH is the relative humidity (%).

**Table 1:** ET, RH, and THI measurements during the experimental period.

Parameter		H	M	L
ET (°C)	08:00–18:00	35	30	22
	18:00–08:00	30	28	20
RH (%)	08:00–18:00	80	85	90
	18:00–08:00	80	85	90
THI	08:00–18:00	91	84	71
	18:00–08:00	83	81	71

ET, Environmental temperature; RH, Relative humidity; THI, Temperature–humidity index.

All Dazu black goats were housed in single pens located in a barn on the Rongchang Campus of Southwest University and fed a total mixed ration (TMR) twice daily at 08:30 and 18:00 h with feed intake *ad libitum* and had free access to clean drinking water. It had displayed the composition and nutrient content of the daily TMR in Table 2. The ME of each raw material was calculated according to the Feeding Standard of Meat-Producing Sheep and Goats of Chinese Agricultural Industry Standards (HB, NY/T 816-2004). The quality of the TMR was checked periodically during the experiment to ensure the absence of mold and spores. The level of aflatoxin in the maize used, as determined by Total aflatoxin detection kit, was below the maximum tolerance threshold in the European Union.

**Table 2:** Ingredient and nutrient composition of the TMR (dry matter basis) %.

Ingredients		Nutrient composition^2^	
Pennisetum sinese Roxb	50.00	DM^3^	87.75
Corn	32.27	ME^4^ (MJ/kg)	10.71
Wheat bran	7.20	CP^5^	11.43
Soybean meal	6.93	NDF^6^	50.56
NaCl	0.50	ADF^7^	36.06
CaHPO_3_·2H_2_O	1.30	Ash	11.05
Limestone	0.80	Ca^8^	0.87
Premix^1^	1.00	P^9^	0.36
Total	100.00		

^1^Per kg of concentrated premix: vitamin A, 45,000 IU; vitamin D, 4,000 IU; vitamin E, 55 mg; vitamin PP, 130 mg; vitamin B_1_, 14 mg; vitamin B_2_, 12.5 mg; vitamin B_6_, 5 mg; vitamin B_12_, 0.02 mg; folic acid, 0.2 mg; Co, 1 mg; Fe, 100 mg; I, 2.5 mg; Mn, 65 mg; Cu, 13 mg; Zn, 225 mg; Se, 0.14 mg.

^2^Metabolizable energy levels were predicted and the rest nutrient levels were measured.

^3^DM, Dry matter; ^4^ME, Metabolizable energy; ^5^CP, Crude protein; ^6^NDF, Neutral detergent fiber; ^7^ADF, Acid detergent fiber; ^8^Ca, Calcium; ^9^P, Phosphorus.

### 2.2 THI

**Figure 1:**
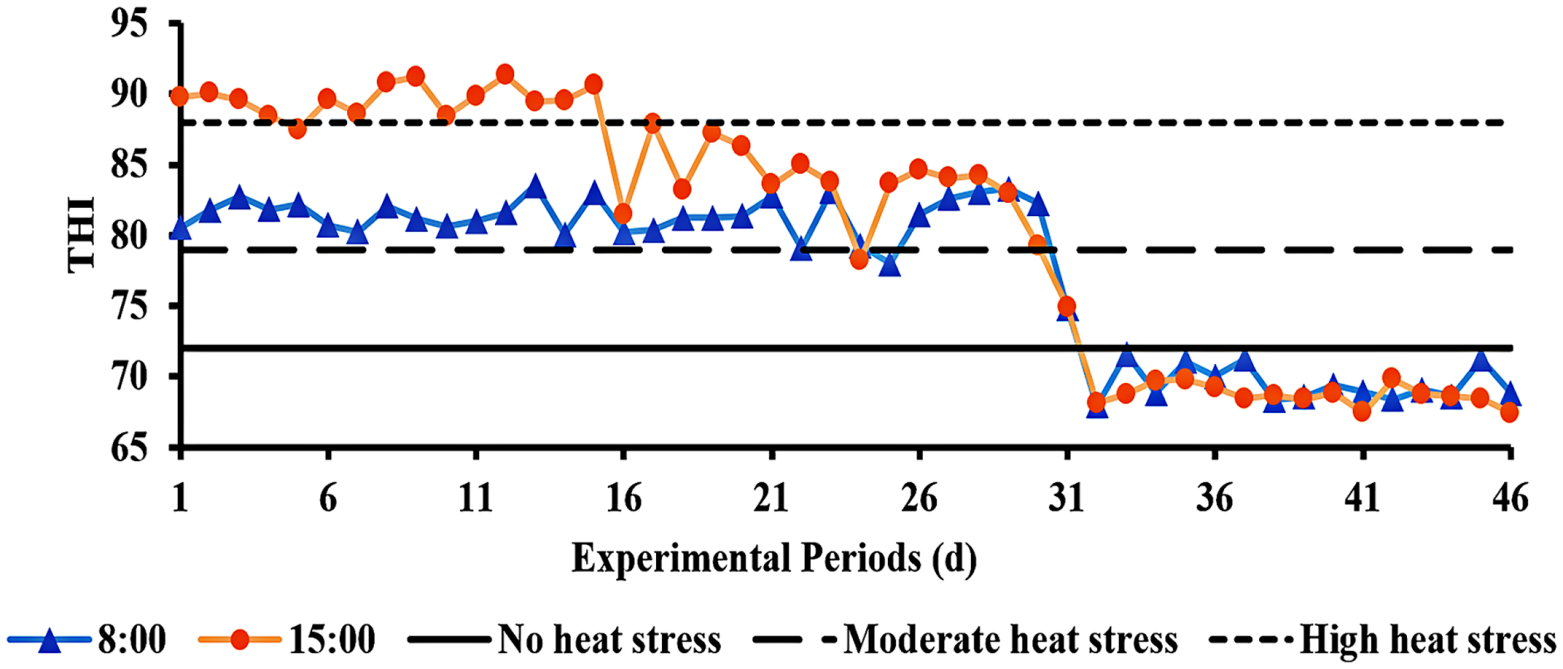
THI of the barn during the experimental period.

As can be seen in Figure 1, during the first period of the experiment, the THI was >88 at 15:00 h, indicating high HS. The THI varied daily from 79 to 88 throughout the experiment’s second period, indicating moderate HS. The THI was <72 in the third experiment period, which meant there was no HS.

### 2.3 Sample collection and analysis

#### 2.3.1 DMI, RT, and RR

The average daily dry matter intake (DMI) of Dazu black goats was calculated as the difference between the initial amount of feed and the amount of feed leftover the following morning.

In accordance with the method described by Tucker et al. (19), rectal temperature (RT) (obtained with a GLA 525/550 Hi-Performance Digital Thermometer, San Luis Obispo, CA) was measured by inserting a disinfected thermometer into the rectum of each goat at 08:00, 14:00, and 18:00 h on d 7 and 15 of each experimental period. The respiratory rate (RR) of each goat was recorded by calculating as breaths/min using a stopwatch for 1 min and the average value of three consecutive measurements was recorded.

#### 2.3.2 Blood biochemistry

Blood samples were collected on d 15 of each experimental period before feeding at 8:00 h and used to prepare serum and plasma samples. After centrifugation at 3,000 rmp for 15 min, the supernatant was aspirated, and aliquots were stored in 1.5-mL centrifuge tubes at −20°C until assayed. Blood glucose (GLU), blood urea nitrogen (BUN), triglycerides (TG), cholesterol (CHO), high-density lipoprotein (HDL-C), and low density-lipoprotein (LDL-C) were measured with an automatic biochemical analyzer (model AU5800; Beckman Coulter, Inc., Brea, CA, USA). Commercial kits were used for the measurement of Non-esterified fatty acid (NEFA) (Wako Chemicals GmbH, Neuss, Germany). Serum levels of total protein (TP), cortisol (COR), triiodothyronine (T_3_), thyroxin (T_4_), and insulin (INS) were measured with enzyme-linked immunosorbent assay (ELISA) kits (Nanjing Jiang Cheng Bioengineering Institute, Nanjing, China).

#### 2.3.3 GTT and ITT

At 08:00 h on d 7 and 10 of each period, GLU tolerance test (GTT) and INS tolerance test (ITT) of all goats was conducted after fasting for 12 h, respectively. A 50% dextrose (0.5 g/kg) solution (AGRIpharm Products, Grapevine, TX) was administered via the jugular catheter and immediately chased with 12 mL of sterile saline. GLU concentration was measured before insulin injection, which was administered at 0.75 U/Kg body weight. Blood samples were collected at 5 min before, at the time of GLU administration (0 min was used as a baseline parameter), and at 5, 10, 15, 20, 30, 45, 60, and 90 min after GLU load. Samples were collected into disposable glass culture tubes containing 250 U of sodium heparin and were immediately placed on ice. And then centrifuged at 3,000 rmp for 15 min. The plasma was divided into 2 aliquots, which were both frozen at −20°C; one aliquot was later analyzed for plasma glucose levels and the other for plasma insulin concentrations.

The GLU and INS responses to the GTT were measured as area under the curve (AUC). Mean GLU values from the samples obtained before the start of a challenge were used as the baseline metabolite concentrations, and the 0 time point sample was used for the INS baseline value. The GLU and INS AUC were calculated through the 90 min sample during the GTT. The blood GLU AUC was calculated through the 90 min sample during the ITT.

#### 2.3.4 Blood metabolites

At 15:00 h, on the last day of all three experimental stages, 3 mL of blood were collected from each goat into tubes coated with heparin sodium as an anticoagulant. Following a 15-min centrifugation at 3,000 rmp, the serum was moved to a fresh tube and kept at −80°C for non-targeted metabolomics examination. Blood samples stored at −80°C were thawed slowly at 4°C for sample pretreatment and analyzed by the Agilent 1,290 Infinity LC ultra-high performance liquid chromatography system (UHPLC). The samples were separated by UHPLC and analyzed by a Triple TOF 5600 mass spectrometer (AB SCIEX). The positive ion (ESI+) and negative ion (ESI-) modes were used for detection, and the raw data were converted into MZXML format by ProteoWizard for data processing.

Non-targeted metabolomics analysis included ultra-high performance liquid chromatography with quadrupole time-of-flight mass spectrometry and pathway enrichment analysis in reference to the Kyoto Encyclopedia of Genes and Genomes (KEGG) database.^1^ The KEGG database and MetPA software were used for pathway analysis of potential biomarkers to identify related metabolic pathways. The KEGG pathway enrichment analysis of differential metabolites was performed by Fisher’s exact test.

#### 2.3.5 Chemical analysis

The TMR samples were dried in an oven at 65°C until a constant weight was achieved. Upon drying, the samples were ground and passed through a 1-mm sieve for further analysis. The chemical composition of dry matter (DM), crude protein (CP), ether extract (EE), ash, calcium (Ca), and phosphorus (P) were measured according to the Association of Official Analytical Chemists (20). Neutral detergent fiber (NDF) and acid detergent fiber (ADF) were determined according to Vansoest et al. (21). The ingredients and chemical composition of the Dazu black goats’ TMR are presented in Table 2.

#### 2.3.6 Statistical analysis

Excel 2016 (Microsoft Corporation, Redmond, WA, USA) was used to record the initial DMI, RT, RR, and blood biochemical data. IBM SPSS Statistics for Windows, version 26.0, was used to analyze the data using one-way analysis of variance or the paired sample t-test (IBM Corporation, Armonk, NY, USA). Graphpad Prism 8 software was used to plot DMI, RT, RR, and tolerance texts graphs (San Diego, CA, United States). Statistical analysis between multiple groups of repeated measurement samples was performed by Repeat ANOVA Meacureme of variance and *p* value was calculated. Results were shown as the Mean ± SD. Probability (*p*) value <0.05 was considered significant. Metabolites with VIP > 1 and *p* < 0.05 that were applied to Student’s *t*-test at univariate level among the groups were considered statistically significant.

## 3 Results

### 3.1 DMI

**Figure 2:**
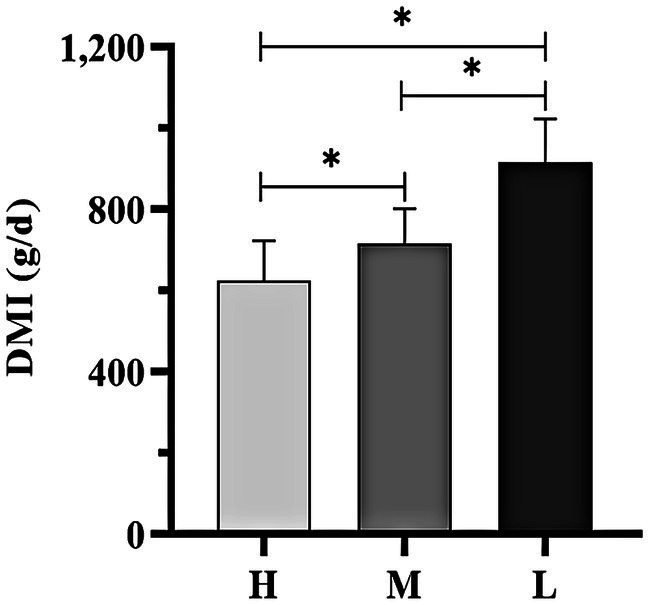
DMI of Dazu black goats of all experimental groups. * indicated significant difference (*p* < 0.05).

The average DMI of Dazu black goats significantly increased as the THI decreased. Notably, DMI was significantly lower in groups H and M as compared to group L (Figure 2, *p* < 0.05).

### 3.2 RT and RR measurements

Table 3 displays the statistical data on RT and RR. Compared to group L HS increased the RT of Dazu black goats at all three time points in groups H and M (*p* < 0.05). But there was no significant difference in RT between groups H and M at 08:00 and 18:00 h (*p* > 0.05). HS significantly increased the RR of Dazu black goats at all three time points (*p* < 0.05). The highest measurements of both RT and RR occurred at 14:00 h.

**Table 3:** RT and RR of all experimental groups.

Times	Groups	RT (°C)	RR (breaths/min)
08:00	H	38.39 ± 0.17^a^	68.17 ± 9.46^a^
	M	38.37 ± 0.21^a^	60.35 ± 7.31^b^
	L	38.10 ± 0.30^b^	14.50 ± 2.78^c^
14:00	H	39.21 ± 0.29^a^	119.17 ± 21.80^a^
	M	38.64 ± 0.19^b^	78.85 ± 11.34^b^
	L	38.38 ± 0.25^c^	14.22 ± 2.10^c^
18:00	H	38.71 ± 0.14^a^	93.83 ± 20.21^a^
	M	38.67 ± 0.16^a^	74.65 ± 7.81^b^
	L	38.32 ± 0.22^b^	14.24 ± 2.51^c^

RT, Rectal temperature; RR, Respiratory rate.

^a, b, c^Demonstrate a statistically significant distinction, *p* < 0.05.

### 3.3 Blood biochemical indices

#### 3.3.1 Blood indices

Compared to group L, HS increased NEFA, COR, and TP concentrations and decreased INS concentration (*p* < 0.05). The BUN concentration increased and the GLU concentration decreased in group H compared to group L (*p* < 0.05). There were no significant differences in CHO, HDL-C, LDL-C, TG, T_3_, and T_4_ contents among the three groups (*p* > 0.05) (Table 4).

**Table 4:** Effects of HS on blood biochemical indices in Dazu black goats.

Project	H	M	L
CHO (mmol/L)	2.39 ± 0.39	2.37 ± 0.59	2.16 ± 0.42
HDL-C (mmol/L)	1.46 ± 0.24	1.39 ± 0.26	1.33 ± 0.21
LDL-C (mmol/L)	0.74 ± 0.14	0.74 ± 0.27	0.60 ± 0.18
TG (mmol/L)	0.39 ± 0.09	0.38 ± 0.07	0.40 ± 0.09
NEFA (μmol/L)	97.53 ± 8.97^a^	90.75 ± 14.04^a^	76.46 ± 10.37^b^
T_3_ (ng/mL)	5.85 ± 0.90	5.80 ± 1.29	6.43 ± 1.17
T_4_ (nmol/L)	131.47 ± 20.1	142.11 ± 42.71	146.15 ± 34.24
COR (μg/dL)	247.69 ± 56.25^a^	208.22 ± 21.79^b^	160.12 ± 41.96^c^
INS (pmol/L)	21.29 ± 0.41^a^	22.06 ± 1.26^a^	31.57 ± 33.68^b^
GLU (mmol/L)	3.65 ± 0.26^a^	4.21 ± 0.69^b^	4.25 ± 0.64^b^
TP (mg/mL)	45.94 ± 3.93^a^	40.22 ± 5.09^b^	26.83 ± 4.70^c^
BUN (mmol/L)	6.23 ± 1.02^a^	6.00 ± 0.93^ab^	5.14 ± 0.98^b^

CHO, Cholesterol; HDL-C, High-density lipoprotein; LDL-C, Low density-lipoprotein; TG, Triglycerides; NEFA, Non-esterified fatty acid; T_3_, Triiodothyronine; T_4_, Thyroxin; COR, Cortisol; INS, Insulin; GLU, Blood glucose; TP, Total protein; BUN, Blood urea nitrogen.

^a, b, c^Demonstrate a statistically significant distinction, *p* < 0.05.

#### 3.3.2 GTT

**Figure 3:**
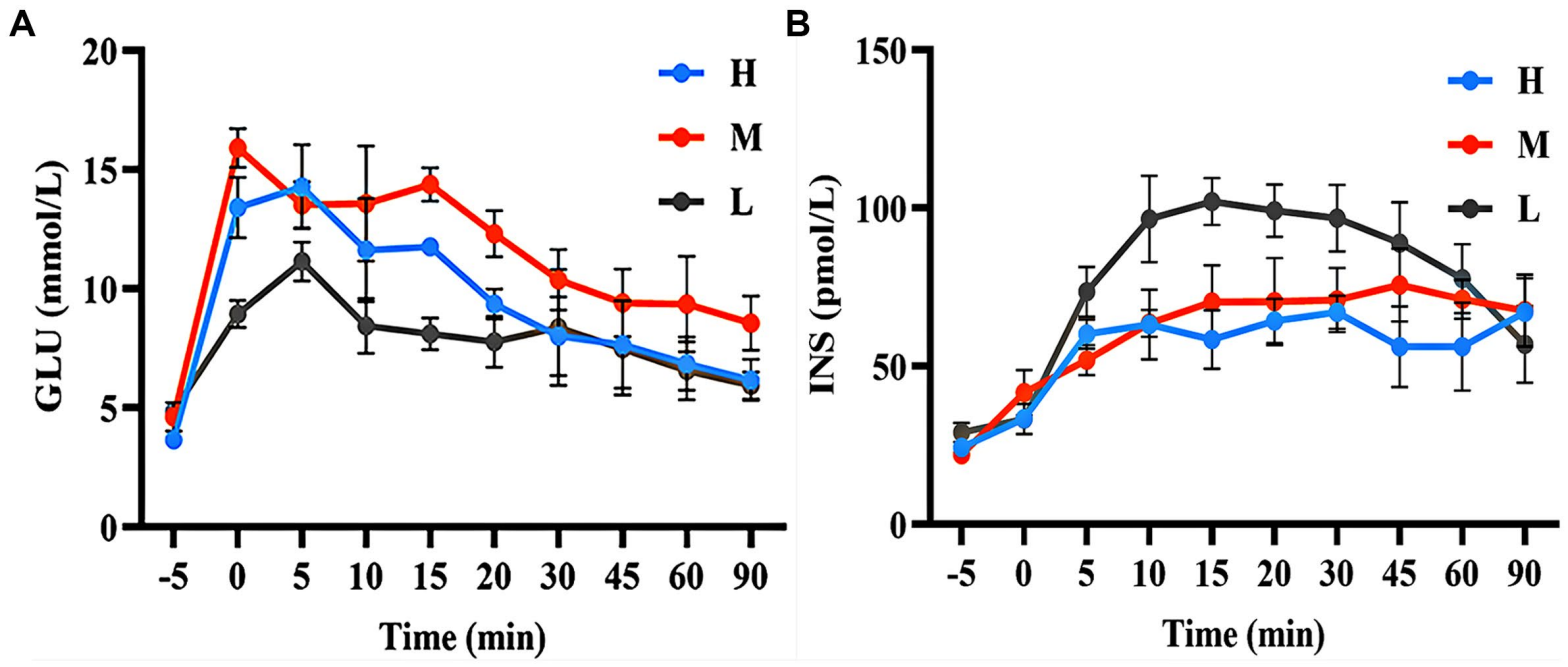
GLU concentration **(A)** and INS concentration **(B)** during the GTT.

As shown in Figure 3A, during the GTT, the GLU concentration in group H was higher than that in group L (20.25–58.00%) during the 0–20 min period. The GLU concentration in group M was higher than that in group L (23.89–77.47%) during the 0–90 min period. Figure 3B shows that the INS concentrations in group H (18.19–42.73%) and group M (8.46–34.17%) were lower than those in group L during 5–60 min.

**Figure 4:**
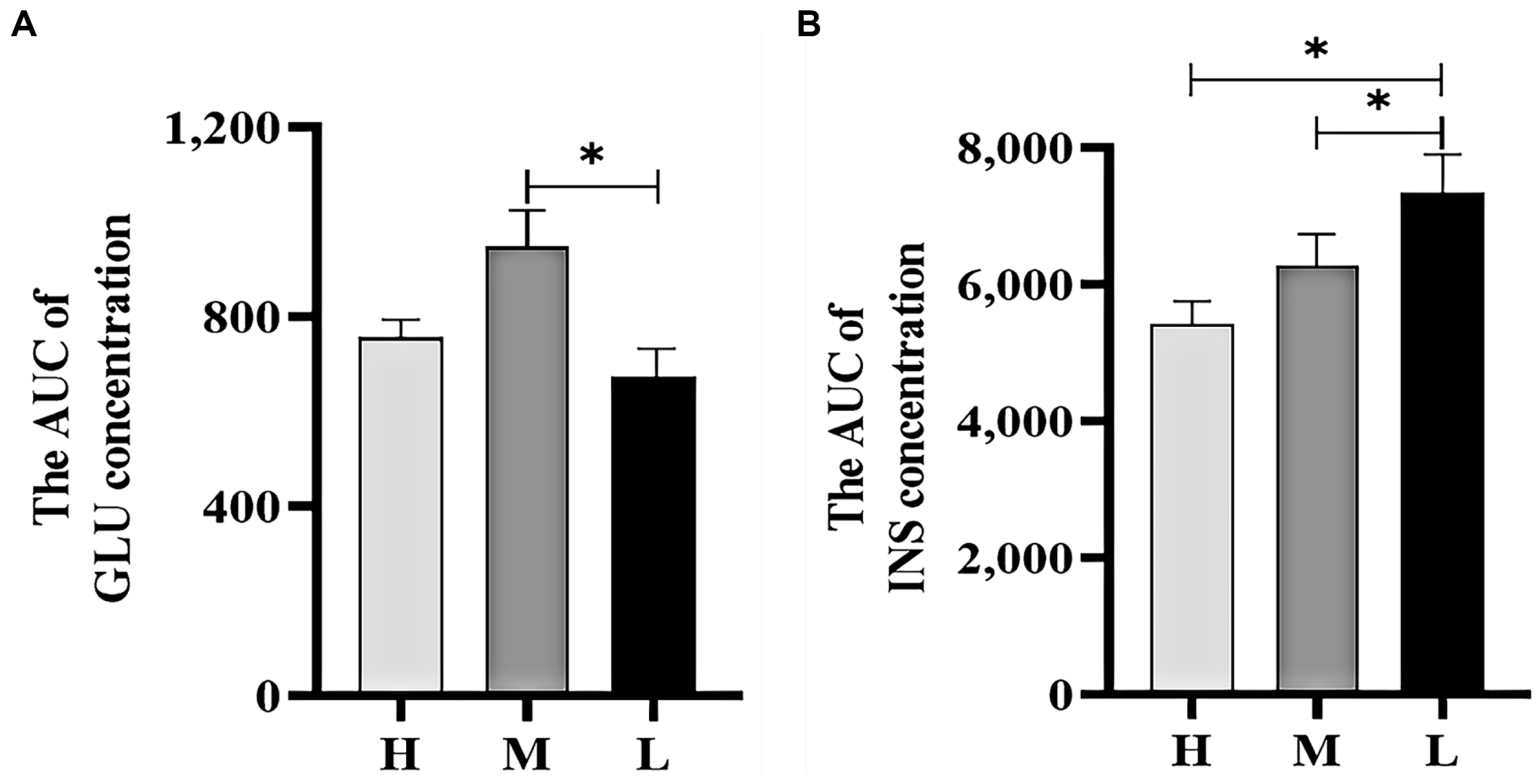
The AUC of GLU concentration **(A)** and INS concentration **(B)** during the GTT. * indicated significant difference (*p* < 0.05).

Figure 4A shows that the AUC of GLU concentrations in groups H and M were 12.26% (*p* > 0.05) and 40.78% (*p* < 0.05) higher than that in group L, respectively. Figure 4B shows that the AUC of INS concentrations in groups H and M were 26.04 and 14.41% (*p* < 0.05) lower than that in group L, respectively.

#### 3.3.3 ITT

**Figure 5:**
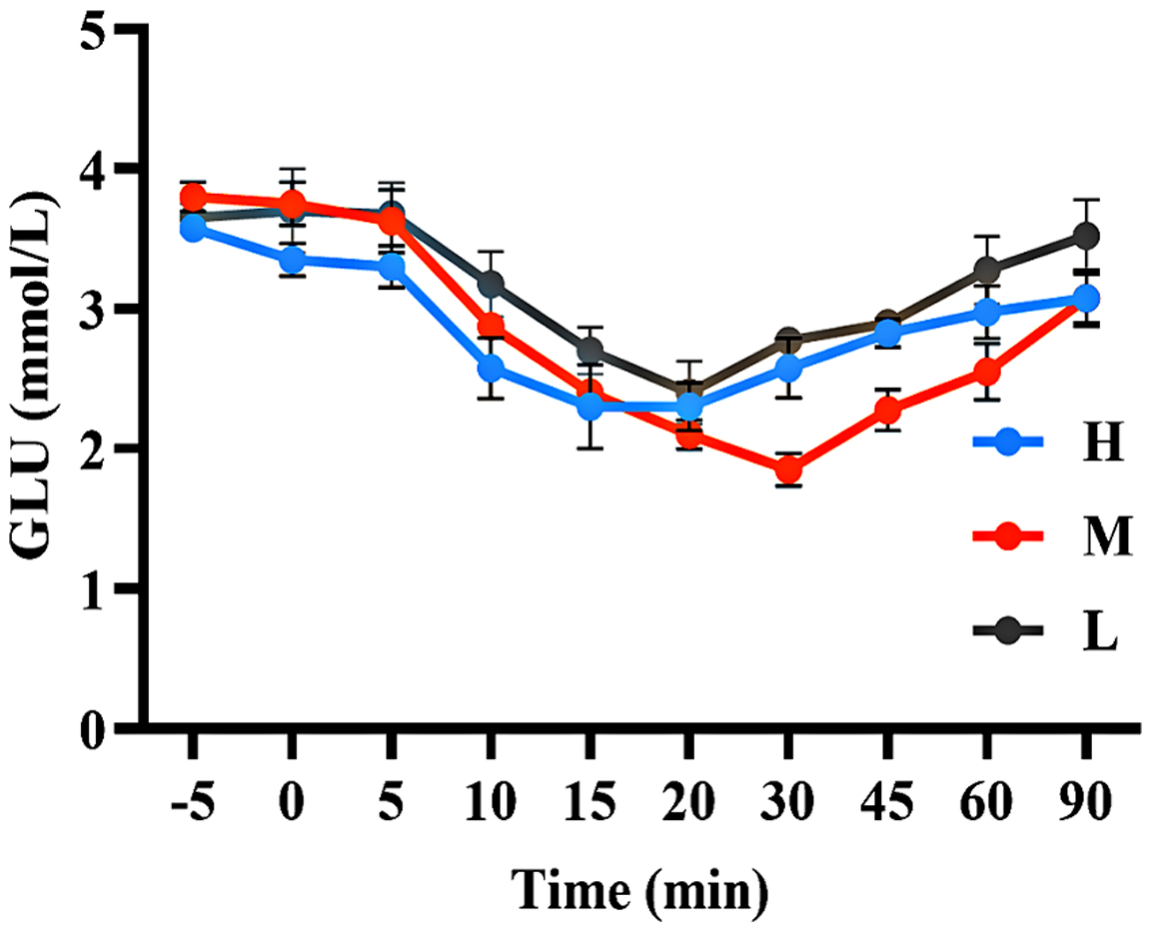
The GLU concentration during the ITT.

**Figure 6:**
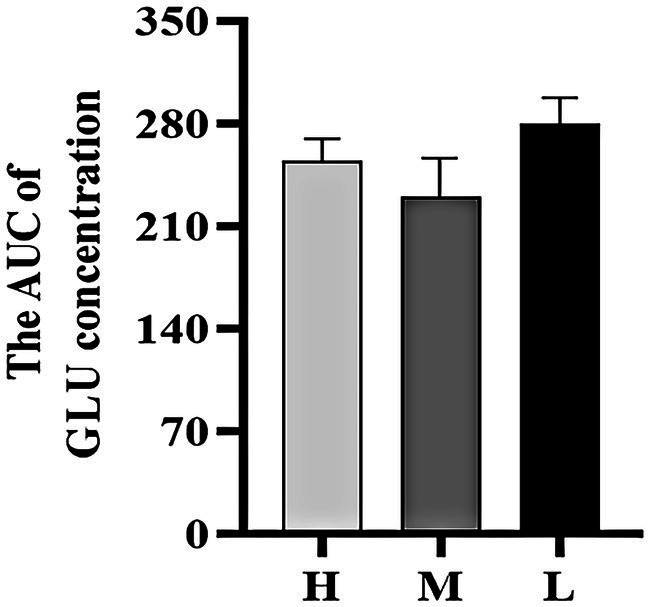
The AUC of GLU concentration during the ITT.

As shown in Figures 5, 6, the AUC of GLU concentrations were not significant among the three groups (*p* > 0.05).

### 3.4 The blood metabolome

**Figure 7:**
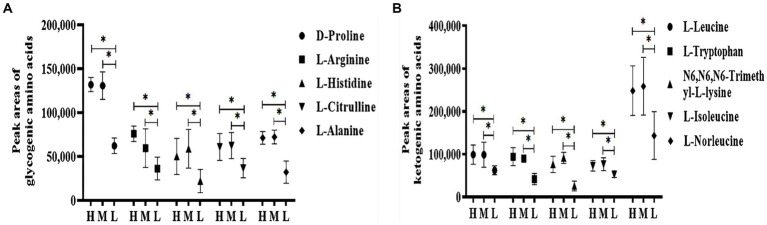
Effects of HS on peak areas of amino acid in Dazu black goats. **(A)** Glycogenic amino acids in the plasma. **(B)** Ketogenic amino acids in the plasma. *indicated significant difference (*p* < 0.05).

In total, there were 75 differential metabolites between groups H and L, 77 between groups M and L, and 26 between groups H and M. As compared to group L, 60 significantly different metabolites were identified in groups H and M, which mainly included amino acids, organic amines, carbohydrates, organic acids, and esters (Table 5). As shown in Figure 7, serum levels of glycogenic amino acids and ketogenic amino acids were significantly increased in groups H and M compared to group L (*p* < 0.05). In addition, serum levels of taurine, creatinine, choline, indole-2-carboxylic acid, L-carnosine, diethanolamine, inosine, creatine, and carbohydrates were also significantly increased (*p* < 0.05), while the serum levels of phenylacetyl glycine, erucamide, alpha-tocopherol, 1-palmitoyl-sn-glycero-3-phosphocholine, creatine, PC (16:0/16:0), 1,2-dioleoyl-sn-glycero-3-phosphatidylcholine, glycerophosphocholine, thioetheramide-PC, indoxyl sulfate, prostaglandin F3α, arachidic acid, and perindopril were significantly decreased (*p* < 0.05) in groups H and M compared to group L.

**Table 5:** Identified differential metabolites of the groups H, M, and L in positive and negative ion modes.

Metabolite	H/L					M/L				
	VIP	FC	*p*-value	Trend	ESI	VIP	FC	*p*-value	Trend	ESI
D-proline	2.63	2.12	2.68E-07	↑	+	2.46	2.27	2.3E-05	↑	+
D-mannose	1.84	3.00	4.66E-07	↑	+	1.40	2.54	0.0006	↑	+
Taurine	2.02	3.03	1.86E-06	↑	+	1.89	3.33	6.45E-05	↑	+
Creatinine	7.89	1.88	9.85E-06	↑	+	6.82	1.83	4.29E-06	↑	+
Choline	3.68	1.66	2.8E-05	↑	+	2.63	1.47	0.0009	↑	+
Indole-2-carboxylic acid	1.96	4.12	3.63E-05	↑	+	1.59	3.64	0.0003	↑	+
Phenylacetyl glycine	2.93	0.30	5.34E-05	↓	+	2.54	0.43	0.0002	↓	+
L-carnosine	1.88	2.04	5.49E-05	↑	+	1.81	2.34	0.0006	↑	+
Diethanolamine	1.59	236.01	6.45E-05	↑	+	1.51	254.49	0.0005	↑	+
Inosine	1.56	2.13	8.36E-05	↑	+	1.43	2.29	0.0046	↑	+
Triethanolamine	1.99	21.28	0.0002	↑	+	1.49	15.43	0.0044	↑	+
L-phenylalanine	2.51	2.05	0.0003	↑	+	2.42	2.17	8.89E-05	↑	+
L-arginine	1.85	2.09	0.0004	↑	+	1.51	2.15	0.0432	↑	+
Erucamide	7.34	0.36	0.0004	↓	+	6.55	0.49	0.0048	↓	+
N6,N6,N6-trimethyl-L-lysine	2.17	2.99	0.0005	↑	+	2.35	3.69	1.58E-05	↑	+
Alpha-tocopherol (vitamin E)	2.30	0.41	0.0005	↓	+	2.12	0.43	0.0027	↓	+
1-Palmitoyl-sn-glycero-3-phosphocholine	3.27	0.77	0.0006	↓	+	3.64	0.71	0.0359	↓	+
Betaine	2.11	2.20	0.0006	↑	+	2.15	2.58	0.0046	↑	+
L-NG-monomethylarginine	1.37	2.99	0.0006	↑	+	1.22	2.83	3.26E-05	↑	+
L-tryptophan	2.12	2.26	0.0008	↑	+	2.02	2.34	4.66E-05	↑	+
Creatine	2.74	0.85	0.0010	↓	+	3.63	0.80	0.0851	↓	+
DL-indole-3-lactic acid	2.22	2.21	0.0014	↑	+	2.20	2.29	6.25E-06	↑	+
Tyramine	1.64	2.05	0.0029	↑	+	1.74	2.31	0.0002	↑	+
L-carnitine	3.46	2.98	0.0044	↑	+	2.75	2.47	0.0004	↑	+
PC (16:0/16:0)	5.12	0.30	0.0059	↓	+	4.74	0.38	0.0169	↓	+
(3-Carboxypropyl) trimethylammonium cation	2.36	1.90	0.0060	↑	+	2.40	2.09	0.0008	↑	+
NG,NG-dimethyl-L-arginine	3.04	2.64	0.0073	↑	+	3.62	3.04	5.26E-05	↑	+
Hypoxanthine	1.83	1.97	0.0080	↑	+	1.28	1.58	0.0414	↑	+
L-leucine	1.68	1.59	0.0081	↑	+	1.84	1.85	0.0085	↑	+
L-isoleucine	1.23	1.39	0.0089	↑	+	1.44	1.64	0.0048	↑	+
N6-methyl-L-lysine	2.43	2.94	0.0108	↑	+	2.91	3.81	0.0011	↑	+
Trimethylamine N-oxide	1.64	5.54	0.0127	↑	+	1.86	7.60	0.0012	↑	+
1-Methylhistidine	2.89	2.52	0.0142	↑	+	1.27	1.95	0.0425	↑	+
L-histidine	1.01	2.46	0.0178	↑	+	1.01	2.85	0.0225	↑	+
L-citrulline	1.42	1.66	0.0241	↑	+	1.20	1.68	0.0139	↑	+
1,2-Dioleoyl-sn-glycero-3-phosphatidylcholine	9.41	0.20	0.0313	↓	+	5.09	0.37	0.0039	↓	+
Cyclohexylamine	3.90	2.69	0.0326	↑	+	3.92	2.69	0.0451	↑	+
L-histidine	1.31	2.28	0.0383	↑	+	1.34	2.45	0.0193	↑	+
Glycerophosphocholine	1.28	1.21	0.0418	↓	+	1.24	3.47	8.02E-06	↓	+
Thioetheramide-PC	3.54	0.38	0.0557	↓	+	3.71	0.34	0.0483	↓	+
Acetyl carnitine	3.91	1.40	0.0687	↑	+	4.65	1.57	0.0338	↑	+
Hippuric acid	4.16	1.59	0.0002	↑	−	3.39	1.53	0.0178	↑	−
L-alanine	1.37	2.20	0.0003	↑	−	1.50	2.36	0.0001	↑	−
Indoxyl sulfate	5.83	0.44	0.0004	↓	−	5.68	0.50	0.0011	↓	−
Salicylic acid	1.99	2.16	0.0006	↑	−	1.53	1.78	0.0041	↑	−
Prostaglandin F3α	7.78	0.35	0.0007	↓	−	7.61	0.41	0.0042	↓	−
3,4-Dihydroxybenzoate (protocatechuic acid)	1.29	2.19	0.0011	↑	−	1.48	2.44	0.0001	↑	−
L-glutamate	1.33	2.53	0.0013	↑	−	1.51	3.32	0.0533	↑	−
15-Keto-PGE1	2.55	5.07	0.0030	↑	−	2.22	4.29	0.0056	↑	−
L-gluonic gamma-lactone	3.17	2.52	0.0032	↑	−	2.29	1.95	0.0914	↑	−
Pseudouridine	1.12	2.31	0.0033	↑	−	1.22	2.39	0.0016	↑	−
Thymidine	1.52	1.85	0.0060	↑	−	1.40	1.82	0.0022	↑	−
Arachidic acid	1.79	0.59	0.0113	↓	−	1.86	0.58	0.0137	↓	−
Allantoin	2.12	1.49	0.0120	↑	−	2.47	1.60	0.0180	↑	−
Taurolithocholic acid	1.93	2.73	0.0144	↑	−	1.83	2.70	0.0023	↑	−
Alpha-D-GLU	6.06	2.39	0.0152	↑	−	7.73	2.89	0.0015	↑	−
L-norleucine	1.83	1.73	0.0155	↑	−	2.56	2.05	0.0048	↑	−
Perindopril	3.99	0.42	0.0271	↓	−	3.53	0.50	0.0421	↓	−
D-allose	1.57	1.78	0.0337	↑	−	1.68	1.89	0.0134	↑	−
DL-lactate	3.57	1.73	0.0352	↑	−	1.15	1.69	0.0457	↑	−

↑, increase; ↓, decrease.

A positive Fold Change (FC) Analysis value indicates an increase, while a negative value indicates a decrease.

VIP, Variable Importance for the Projection.

*P* < 0.05 was considered to indicate significant difference.

Comprehensive analysis of the differential metabolites obtained by comparisons of two pairs using KEGG metabolic pathways. The results showed that the differential metabolites in plasma were associated with “amino acid metabolism,” “lipid metabolism,” “carbohydrate metabolism,” “nucleotide metabolism,” “central carbon metabolism,” “aminoacyl-tRNA biosynthesis,” “ABC transporter” as well as digestion and absorption of proteins and minerals (Table 6).

**Table 6:** KEGG pathways affected by HS in Dazu black goats.

KEGG level 1	KEGG level 2	KEGG level 3	Rich factor	*p*	Rich factor	*p*
			H/L		M/L	
Metabolism	Amino acid metabolism	Histidine metabolism	0.106383	7.27E-05	0.106383	8.61E-05
		Phenylalanine metabolism	0.069444	0.0006	0.069444	0.000652
		Arginine and proline metabolism	0.064935	0.0008	0.064935	0.000886
		Glycine, serine, and threonine metabolism	0.08	0.0012	0.08	0.001382
		Arginine biosynthesis	0.130435	0.0013	0.173913	6.47E-05
		Phenylalanine, tyrosine, and tryptophan biosynthesis	0.085714	0.0043	0.085714	0.004719
		Valine, leucine, and isoleucine biosynthesis	0.086957	0.019698	0.086957	0.021044
		Alanine, aspartate and glutamate metabolism	0.071429	0.028557	0.107143	0.00248
	Metabolism of other amino acids	Taurine and hypotaurine metabolism	0.136364	0.0011	0.181818	5.39E-05
		beta-Alanine metabolism	0.0625	0.036581	0.0625	0.039004
		D-Glutamine and D-glutamate metabolism			0.166667	0.005883
	Lipid metabolism	Glycerophospholipid metabolism	0.096154	0.0001	0.096154	0.000141
		Linoleic acid metabolism	0.107143	0.0022	0.071429	0.030475
		Secondary bile acid biosynthesis			0.055556	0.048326
		Primary bile acid biosynthesis			0.06383	0.010758
		Biosynthesis of unsaturated fatty acids	0.055556	0.014269		
	Carbohydrate metabolism Energy metabolism	Fructose and mannose metabolism	0.055556	0.014269	0.055556	0.015681
		Methane metabolism	0.035714	0.044956		
		Nitrogen metabolism			0.105263	0.014585
	Biosynthesis of other secondary metabolites	Glucosinolate biosynthesis	0.053333	0.0054	0.053333	0.006082
		Tropane, piperidine and pyridine alkaloid biosynthesis			0.044118	0.028777
	Nucleotide metabolism	Pyrimidine metabolism			0.045455	0.026639
Genetic Information Processing	Translation	Aminoacyl-tRNA biosynthesis	0.153846	2.14E-08	0.192308	4.31E-11
Environmental Information Processing	Membrane transport	ABC transporters	0.09375	1.6E-09	0.117188	6.53E-13
	Signal transduction	mTOR signaling pathway	0.333333	0.028205	0.333333	0.029203
		FoxO signaling pathway	0.2	0.046573	0.2	0.048204
		Two-component system			0.053571	0.017285
Cellular Processes	Transport and catabolism	Lysosome	0.25	0.037432	0.25	0.038749
Organismal Systems	Digestive system	Protein digestion and absorption	0.191489	3.3E-10	0.234043	4.06E-13
		Mineral absorption	0.172414	6.41E-06	0.206897	2.76E-07
		Bile secretion			0.028571	0.027977
	Nervous system	Retrograde endocannabinoid signaling	0.157895	0.0007	0.105263	0.014585
		Long-term depression	0.222222	0.0031		
		Glutamatergic synapse			0.25	0.00256
		GABAergic synapse			0.222222	0.00327
	Excretory system	Proximal tubule bicarbonate reclamation	0.117647	0.01098	0.176471	0.000556
Human Diseases	Cancers: Overview	Central carbon metabolism in cancer	0.216216	1.22E-09	0.243243	4.52E-11
		Choline metabolism in cancer	0.272727	0.0001	0.272727	0.000141
	Neurodegenerative diseases	Amyotrophic lateral sclerosis (ALS)	0.2	0.0038	0.2	0.004062
		Huntington disease	0.333333	0.028205	0.333333	0.029203
	Infectious diseases: Bacterial	Salmonella infection	0.25	0.037432	0.25	0.038749
	Infectious diseases: Parasitic	Amoebiasis	0.153846	0.006452		

*P* < 0.05 was considered to indicate significant difference.

## 4 Discussion

### 4.1 Physiological indicators

In this experiment, DMI increased as the THI decreased. In response to HS, animal secretion and expression of adiponectin and leptin will increase, adiponectin regulates feeding behavior by stimulating peripheral receptors to transmit nerve impulses to the hypothalamus, while leptin stimulates the hypothalamic appetitive center to reduce food intake (22–25), ultimately reducing metabolism and heat production. Reduced DMI and nutrient digestibility by HS can lead to undernutrition in animals and adversely affect their health (26). HS is reported to decrease DMI in sheep (27), dairy goats (18), and dairy cows (28).

RR and RT are the most common physiological indices of HS. Exposure to high ET inhibits the ability to dissipate heat, resulting in increased RT. Meanwhile, the RR is increased to enhance lung ventilation and dissipate heat (29). Under suitable environmental conditions, the RT of ewes fluctuates between 38.3°C and 39.0°C (30). Shilja et al. (31) found that the RR and RT of goats were significantly higher in the HS group than in the non-HS group (69.17 breaths/min and 39.08°C vs. 31.92 breaths/min and 38.70°C, respectively). Marai et al. (32) reported that the RR and RT of goats were significantly higher in the summer than the winter, but varied among different breeds, and physiological changes were greater in cold-adapted breeds during the summer than heat-adapted breeds. The results of the present study showed that the RR and RT of goats were significantly increased in response to HS, as RT increased to 39.8°C. Banerjee et al. (33) found that an increase in RT of ≤1°C decreased productivity and reproductive capacity.

### 4.2 Blood biochemistry

The hypothalamic–pituitary–adrenal axis of the neuroendocrine system is primarily involved in the stress response (34). HS-induced stimulation transmits nerve impulses through the cerebral cortex to the hypothalamus, which then releases hormones that promote the secretion of adrenocorticotropic hormone (ACTH) and inhibit the production of thyroid hormone (TSH). ACTH and TSH act on the adrenal and thyroid glands, respectively, thus increasing the secretion of COR by the adrenal glands while decreasing the production of T_3_ and T_4_ by the thyroid gland. In response to HS, serum levels of COR are increased (35). COR is a common biomarker of the stress response and can help maintain the internal environment and reduce HS-induced damage (36, 37). However, excessive production of COR in response to long-term HS can damage immune-related organs by inducing the release of inflammatory factors and promoting the aggregation and adhesion of leukocytes, thereby triggering an inflammatory response. Exposure to excessive heat for more than 2 h will elevate serum COR levels (38). Przemyslaw et al. (39) reported that exposure to ET of 50°C increased serum COR levels of Merino rams by nearly 10 fold. In addition, HS-induced increases in serum levels of COR and epinephrine generally inhibit the production of INS and TG while promoting glycolysis, gluconeogenesis, lipolysis, and the production of NEFA (40, 41).

Low serum GLU can promote lipid mobilization (42), resulting in the release of NEFA into the blood for energy production. Therefore, increased serum levels of NEFA can conserve GLU (28). The decrease in serum levels of GLU with increased concentrations of NEFA in response to HS supports this view. HS was confirmed to decrease serum levels of GLU in cows (43), goats (44), and calves (45). The results of GTT and ITT showed that HS impaired GLU tolerance, but did not change INS tolerance and sensitivity, so it is speculated that HS may change blood GLU concentration by affecting INS secretion rather than sensitivity. In the context of HS, the increase in blood GLU concentration during the GTT can be attributed to the altered metabolic responses induced by the stress condition. HS can lead to the release of stress hormones such as COR, which can promote gluconeogenesis and glycogenolysis, consequently elevating blood GLU concentration (46). Additionally, HS may reduce INS secretion, further contributing to the hyperglycemic response observed during the GTT under HS conditions (47).

HS can promote the production of free radicals and subsequent oxidative damage and apoptosis (48, 49). Oxidation helps to maintain cellular integrity and provides energy. Antioxidation works in tandem with aerobic metabolism to combat free radical-induced tissue damage. Moreover, reduced production of antioxidants can promote oxidative stress (48) and increase secretion of inflammatory cytokines (50). Excessive oxidation induces the body to produce excessive inflammatory response and immune response, which further aggravates the damage of tissues, organs and systems. Reactive oxygen species (ROS) can increase protein degradation while reducing protein synthesis (48). A large number of ROS can damage the protein structure, DNA structure, cell membrane structure and various organelles of cells, thereby causing systemic inflammation, such as fatty liver, laminitis, metritis and mastitis, and reducing the yield and quality of milk and meat. Finocchiaro et al. (51) found that the protein content in ewe milk was negatively correlated with the THI during HS. In the present study, the TP increased significantly under HS, indicating that goat body protein degradation increased at this time.

### 4.3 Blood metabolite

**Figure 8:**
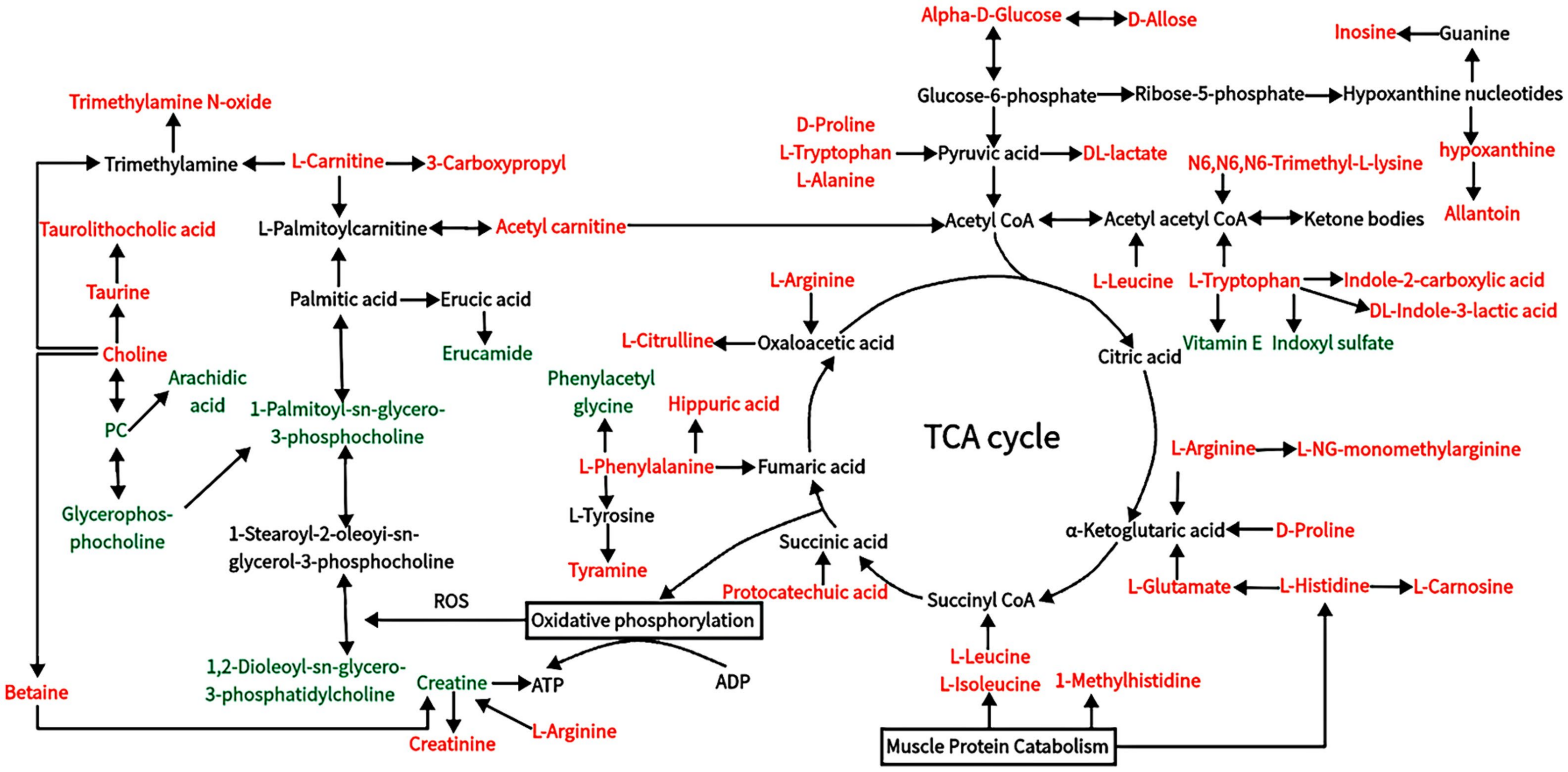
Changes in blood metabolic pathways of Dazu black goats under HS. The metabolites are colored according to the type of change in response to HS (black, no change; red, upregulation; green, downregulation).

In this study, there were notable changes to metabolites of carbohydrate metabolism, gluconeogenesis, and glycolysis (e.g., alpha-D-GLU, D-allose, Phenylacetyl glycine, hippuric acid, glycogenic amino acids, and DL-lactate) (Figure 8 and Table 6), indicating that HS influences the energy metabolism of Dazu black goats. The increased contents of alpha-D-GLU and D-allose in response to HS indicate decreased energy expenditure with increasing ET, which could reduce heat production in goats. Serum levels of glycogenic amino acids, including D-proline, L-arginine, L-histidine, L-citrulline, and L-alanine, were increased in response to HS, indicating enhanced gluconeogenesis. In a state of HS, goats experience decreased DMI and energy supply, leading to insufficient nutrient intake, which results in the use of stored nutrients for energy, thereby increasing protein degradation and serum levels of amino acids. When the energy supply is low, glycogen reserves are limited, and carbohydrate transport occurs through gluconeogenesis (52). In addition, decreased production of TSH, T_3_, and T_4_ with increased production of ROS in response to HS will increase protein degradation and serum levels of amino acids. Guo et al. (53) found increased serum concentrations of total amino acids in cows in response to HS, especially glucogenic amino acids (alanine, aspartic acid, glutamic acid, and glycine). Cowley et al. (54) reported that low serum concentrations of GLU can increase the consumption of amino acids, thereby promoting gluconeogenesis in cows in response to HS. DL-lactate, the main metabolite of glycolysis, accumulated in the blood of Dazu black goats during HS. In addition to the amino acids involved in gluconeogenesis, those related to glycolysis are also increased by HS (55). L-alanine can regulate gluconeogenesis and glycolysis to ensure energy production when energy intake is insufficient (56). Increased serum levels of metabolites, such as alpha-D-GLU, glucogenic amino acids, and DL-lactate, indicate enhanced gluconeogenesis and glycolysis in response to HS to meet energy requirements.

L-carnitine and acetyl carnitine are metabolites of fatty acids (57) and act as carriers of long-chain fatty acids, such as palmitoylcarnitine, across the mitochondrial inner membrane for fatty acid β-oxidation. Acetyl carnitine is produced in the mitochondrial matrix by carnitine and acetyl coenzyme A (58). Metabolites of acetylcarnitine can undergo fatty acid oxidation and enter the tricarboxylic acid cycle. Accumulation of L-carnitine indicates inhibition of β-oxidation in goats in a state of HS. However, L-carnitine can be beneficial by inhibiting aerobic oxidation of lipids, thereby reducing oxidative stress (59). The main application of vitamin E is as an antioxidant to protect polyunsaturated lipids from damage brought on by free radicals (60). In this study, the content of vitamin E was significantly decreased in goats in response to HS, indicating that HS may reduce the production of antioxidants. Therefore, Dazu black goats may resist cellular oxidative stress by reducing fatty acid β-oxidation rather than antioxidant regulation. Under physiological conditions, energy is produced by the complete oxidation of fatty acids in the kidney, myocardium, and other tissues. However, incomplete oxidation of fatty acids in liver cells forms ketone bodies. Notably, serum levels of ketogenic amino acids (L-leucine, L-tryptophan, L-lysine, L-isoleucine, and L-norleucine) were relatively increased in the HS group as compared to the non-HS group. Low serum GLU indicates insufficient intake of exogenous nutrients and cellular energy production. Intake of amino acids by liver cells increases production of ketone bodies, which then enter the circulation and are oxidized in extrahepatic tissues to supply energy. The L-leucine and L-isoleucine not only participate in ketogenesis but also play roles in immune regulation and protein metabolism (61, 62).

In addition to influencing the metabolism of liver cells, HS also significantly impacts the kidneys, which are crucial for physiological functions (63, 64). Under the conditions of HS and decreased feed intake, amino acid utilization is increased, leading to increased methylhistidine production as a marker of muscle fibrinolysis and increased liver urea synthesis (52). Abdelnour et al. (8) and Kamiya et al. (65) found that HS could break down histamine, as evidenced by increased serum concentrations of methylhistidine and urea. In the present study, serum levels of methylhistidine were significantly increased in response to HS, indicating increased fibrin catabolism. HS also increases serum concentrations of catecholamine and COR, leading to increased resistance in the kidneys and visceral vessels, resulting in renal ischemia and the timely excretion of metabolites (66). Furthermore, HS causes vasodilation and evaporative water loss, leading to lower blood pressure, followed by increased water retention, resulting in decreased renal clearance (67, 68). These two conditions are the main reasons for elevated serum concentrations of methylhistidine and urea. In addition, creatinine levels are significantly increased under HS conditions (8). The serum content of creatinine, a metabolite of creatine, is a reliable indicator of muscle tissue degradation (69). The serum concentration of creatinine is dependent on glomerular filtration and increases with impaired renal function. Although the creatine content was reduced under HS conditions due to decreased renal clearance, serum creatinine eventually increased. Citrulline is a product of muscle metabolism and is mainly filtered by the kidneys. Therefore, the serum citrulline concentration is considered a marker of renal function (70). An abnormally high serum citrulline level is an indicator of impaired renal function.

Serum levels of choline, betaine, taurine, and taurocholic acid were significantly increased, suggesting that Dazu black goats mainly recover from HS by producing a series of metabolites. Phospholipids, including glycerophospholipids and sphingosine phospholipids, are the main components of biofilms. In animals, phospholipids are hydrolyzed into glycerol, phosphate, choline, and ethanolamine by a series of phospholipases. Glycerophospholipids are major lipids in cell membranes and play important roles in cell signaling, G protein-coupled receptors, and ion transport (71). In the present study, serum levels of glycerophosphocholine, 1,2-dioleoyl-sn-glycero-3-phosphatidylcholine, and 1-palmitoyl-sn-glycero-3-phosphocholine were decreased in Dazu black goats in response to HS (Figure 8). Decreased levels of glycerophospholipid metabolites, such as hotline, indicate changes to the cell membrane structure and function of Dazu black goats. In addition, a significant decrease in glycerophospholipids alters the permeability and fluidity of cell membranes, which can serve as a defense mechanism to protect against oxidative damage caused by ROS (72, 73). Choline is a key precursor for the synthesis of acetylcholine (74), which can be easily oxidized to betaine (75), and plays an important regulatory role in maintaining cellular structural integrity and reducing oxidative stress (76). Heat shock proteins (HSPs) gradually restore proteins denatured by heat damage to normal states by refolding and preventing protein aggregation. Betaine can effectively improve the folding rate and decomposition rate of HSPs, thus enhancing their ability to withstand HS (77, 78). Choline, which reduces oxidative stress and thermal injury through a series of enzymatic reactions, produces glutathione and taurine (79, 80). Taurine can neutralize cholic acid in the liver to synthesize taurocholate, which promotes the absorption of lipids and fat-soluble vitamins. Chronic HS has been shown to influence purine metabolism, RNA transport, and down-regulate the metabolism of L-arginine and D-proline in dairy goats, while activating pathways associated with apoptosis and inhibiting pathways associated with tissue repair (81). In this study, serum levels of inosine and hypoxanthine were increased in response to HS, indicating that HS can induce apoptosis. Therefore, the contents of choline, betaine, taurine, and taurocholic acid were increased to recover from heat injury, and the increased L-arginine and D-proline may be mainly used for energy metabolism.

## 5 Conclusion

HS decreased DMI and increased the RR and RT in Dazu black goats. HS changed blood hormone levels, increased protein degradation, increased plasma amino acid concentrations, elevated lipid levels, and impaired GLU tolerance. Gluconeogenesis, glycolysis, and ketogenic metabolism are increased under HS, thereby altering energy metabolic pathways in goats. Moreover, Dazu black goats primarily used an increase in amino acid metabolism as a source of energy in HS. In addition, in response to HS, a series of metabolites are produced to restore the heat damage of the organism.

## Data availability statement

The original contributions presented in the study are included in the article/supplementary material, further inquiries can be directed to the corresponding authors.

## Ethics statement

The animal study was approved by the Institutional Animal Care and Use Committee of Southwest University (Chongqing, China). The study was conducted in accordance with the local legislation and institutional requirements.

## Author contributions

LW: Conceptualization, Data curation, Formal analysis, Investigation, Methodology, Validation, Writing – original draft, Writing – review & editing. PZ: Data curation, Investigation, Validation, Writing – original draft. YD: Investigation, Validation, Writing – original draft. CW: Investigation, Validation, Writing – original draft. LZ: —. LY: Conceptualization, Data curation, Formal analysis, Investigation, Methodology, Validation, Writing – original draft. FZ: Conceptualization, Funding acquisition, Project administration, Resources, Writing – review & editing. WH: Conceptualization, Data curation, Formal analysis, Funding acquisition, Project administration, Resources, Writing – review & editing.

## Footnotes

^1^ https://www.genome.jp/kegg/
